# Clinical Remarks on Surgical Cases Occurring at the Buffalo Hospital of the Sisters of Charity

**Published:** 1874-10

**Authors:** Julius F. Miner


ART IV.—*Clinical Remarks upon Surgical Cases occurring at the Buffalo Hospital of the Sisters of Charity.* By Prof. JULIUS F. MINER, M. D. Reported by W. W. MINER, M. D.

I am happy to meet again the familiar faces of so many who have before been students together here. I take great pleasure also in welcoming here for the first time, so many whose faces are not yet familiar, but who are enlisted together with us in professional study and aim.

The physician, old or young, is a constant student of his profession. He is constantly gaining new scientific truths and fresh knowledge in his chosen pursuit. More than this, he is constantly extending and confirming an experience, in the personal observation and practical management of men, as subjects of the various physical ills. A large amount of scientific truth is readily to be gained from the study of books, by lectures and counsel from practical men; experience is of slower attainment, involves more than mere study, and is to that extent more desirable, more valuable.

Here, indeed, is the grand distinction between a young student and an older one, between a young practitioner and a physician of years. The public esteem more highly the services of the former, and for what reason? Years are expected to bring experience to a man, and thus render him more capable in his work than those who are inexperienced. It is not only what the physician knows, it is what he is capable of, that impresses his patients and the public generally. One needs to be conscious of his own capability in order to secure public patronage. Actual experience is necessary to give a man confidence, confidence in himself, in his own ability. Nothing can be a real substitute for it. The practiced physician feels able to discern that which he has before observed, feels competent to take charge of that which he has before cared for.

Clinical experience will be of great value to you when you hereafter engage in the active duties of your profession. You are here afforded opportunities of making your diagnosis in actual cases, of determining the proper procedure with them, and witnessing the results in their management. Some of the cases brought to your notice occur rarely in single communities or in one man's experience; others are of frequent occurrence and will early demand your professional attention. To have once before seen cases and determined concerning them yourself, will render you in a degree capable to recognize and care for them in future.

CASE I.—*Amputation of Fingers.*—Antony L——, aged 23 years, in occupation a farmer, received on the twentieth of September, an injury to his left hand, by the accidental bursting of a gun. He was received into the hospital on the following day, and cared for by Dr. Cronyn, one of my colleagues in hospital service. It was found by him that the soft parts of the thumb of the injured hand were very extensively lacerated, and the extremities of two fingers blown off. The thumb, you notice, has very nicely recovered its integrity. The bony stumps of the second and third fingers are now denuded of integument and project so as to require retrenchment. In the surgery of the fingers it is astonishing to witness the extent of repair that sometimes takes place; the hand seems to possess advantages in this respect over other parts of the body. In amputation, flaps are made of palmar and dorsal integument, and these are approximated with the same care as in amputation of limbs. Disarticulation is preferable on many accounts to amputation in the continuity of a phalanx. No arteries need ever be ligated in operations upon fingers, a little pressure, or the closing of the wound with stitches, will speedily arrest any hemmorrhage that may occur from them.

CASE II.—*Harelip.*—A child of M— K—, aged six months, has as you notice, a cleft upper lip. The fissure, in this case, does not involve the maxillary or palatal bones, as it frequently does in these cases. Ether is given, and the upper lip separated to some extent from its attachments below to the superior maxilla; the edges of the fissure, thoroughly pared, and then carefully approximated by the twisted suture. Hemmorrhage is advantageously controlled by the application of suitable forceps, which grasp and compress the margins of the lip on either side, at the angles of the mouth; or they may be seized by the fingers of assistants. In the application of the silk to the suture pins, it is very necessary that care be used, as too much pressure from this source will strangulate the circulation of the parts included, and cause them speedily to slough away. A piece of adhesive plaster may be applied so as to draw forward the integument on either side of the face, and give support to the sutures.

On October the 14th, at the following clinic, the child was presented, and it was found that during the four days previous, complete union of the wound had taken place. The pins were accordingly removed, an adhesive plaster strip being reapplied. It is desirable that the parts forming the margin of the mouth should be nicely and evenly adjusted, as irregularity here is readily noticeable. If perfect apposition is not at first obtained, in this respect, a subsequent operation could be made that would remedy the fault.

Case III.—*Dislocation of the Elbow.*—Agnes G—, aged 30, fell while engaged in washing windows, and caused an injury which renders her right arm useless. It is now two weeks since the injury was received, and the case is one in whose diagnosis you may take very much interest. The elbow is somewhat swollen, and motion of the joint is painful and limited. On careful examination you may discover, if you are looking for it, that the upper extremity of the olecranon process of the ulna, when the arm is, in a degree, flexed, projects an inch or so farther back than do the projections of the condyles of the humerus. Now by examining this articulation in the skeleton, you will see that the olecranon process should be on an exact line, vertically, with the condyles of the humerus, when the joint is flexed at a right angle. This you will see is not the condition in the patient before us. Failure to recognize dislocation of the ulna backwards, is a most fertile source of suits for malpractice. You will do well to avoid the deplorable results of these cases. The other dislocations occurring in the elbow, present such deformity that they cannot be overlooked. With the diagnostic points clear in your mind, you ought to be able to recognize the backward dislocation, here admirably presented. Sad mistakes are made in the management of such cases, and while the necessity of a proper recognition and treatment of them is very evident and urgent, still I am inclined to regard with indulgence those who have made errors in the treatment of them. I have sometimes found that the swollen condition of the parts in a fleshy person, rendered it a little difficult to find the projections which guide to a decision.

The reduction of this dislocation is accomplished by the use of chloroform and traction at the wrist, with counter-traction at the arm and shoulder, while moderate movement of the joint is made. Extending the forearm out beyond a straight line, is of advantage in releasing the coronoid process from its engagement in the olecranon fossa. After reduction has been accomplished, the arm is placed in a sling and kept in a flexed position.

Reduction in the present case, which is of two weeks duration, is acomplished readily, though requiring considerable force of traction. I have reduced them after three months had elapsed. In one case of that duration I failed: it may be that there had been fracture of the condyles, at any rate the joint rémained permanently a stiff one. These cases may be presented to you when they have been long dislocated. I should be inclined to attempt restoration even after many years had elapsed; would not say that an elbow is irreducible after it has been more than three months dislocated. Try reduction and try it well, before deciding a case is irreducible. In dislocations of the shoulder, though they are reducible at a late date, the glenoid cavity is shallow and easily becomes filled up with inflammatory deposits which render retention ot the head of the humerus uncertain. The elbow is less liable to the occurrence of this obstacle to restoration, as the sigmoid cavity of the ulna could hardly be obliterated in this manner.

A suit of malpractice which had a bearing upon the after-treatment of reduced luxations of the elbow, occurred in this region not long since. A good resolute surgeon supposes that he has put back a dislocated elbow—shows by good respectable fellow-practitioners, that it was put back, and this is confirmed by four or five good observers. Some months afterwards he is sued for malpractice in the case, and the melancholy feature of it is, that all agree the elbow is now out of place. At the trial, the doctors swore as positively as doctors can, that the elbow was reduced, and was all right at one time. Some superior and respectable surgeons from abroad, testified that it would not reluxate spontaneously, and with this ended the first trial. At the second trial one of the same authoritative witnesses swears that since the first trial he has gained new light on the subject of reluxation, has in fact had a case in which reluxation took place; says it will occur spontaneously, and that flexion of the forearm, fully to a right angle, is generally necessary in the after-treatment to prevent it. If the coronoid process of the ulna is fractured, I can see how reluxation might occur when the arm was in a straight position, and that flexion would retain it in its place. If I had a case of reluxation, should presume that the coronoid process was broken off. If this is not the case, and the proper tendons attached to the ulna are in place, I do not believe reluxation will occur.

I have had repeated opportunities to test the liability of the occurrence of reluxation in the elbow, and in no single instance have I been able to obtain this result. I swore positively one year ago, that after reduction the laying of the elbow upon a pillow, when flexed forty five degrees, obtusely, that is half-way between straight and a right angle, was unobjectionable treatment; that it would not then reluxate by any naturally operative forces, and that it was not necessary to flex to any greater extent in the after-treatment of these cases. Flexion beyond a certain degree in the after-treatment of some cases, such as I have noticed, is rendered impracticable by the swollen condition of the joint and the pain which it occasions. These opinions then sworn to in court, I would at present maintain.

